# Draft genome of *Acinetobacter baumannii* strain niger01 co-producing *bla*_NDM-1_ and *bla*_OXA-23_ genes in Niger

**DOI:** 10.1128/mra.01300-25

**Published:** 2025-12-29

**Authors:** Abdourahamane Yacouba, Oumou Hamidou, Ahmed Olowo-Okere, Abdoulaye Ousmane, Massir Issoufou-Noma, Bassirou Abdou-Natali, Ounoussa Tapha, Mahamadou Doutchi, Saidou Mamadou

**Affiliations:** 1 Faculty of Health Sciences, Abdou Moumouni University 107945, Niamey, Niger; 2 National Reference Laboratory for Antimicrobial Resistance, Hôpital National Amirou Boubacar Diallo 667850 , Niamey, Niger; 3 Faculty of Pharmaceutical Sciences, University of Abuja 99399, Abuja, Federal Capital Territory, Nigeria; 4 Faculty of Health Sciences, Dan Dicko Dankoulodo University 665091, Maradi, Niger; 5 Faculty of Health Sciences, Andre Salifou University 553100, Zinder, Niger; University of Pittsburgh School of Medicine, Pittsburgh, Pennsylvania, USA

**Keywords:** *Acinetobacter baumannii*, *bla*_NDM-1_, antimicrobial resistance, Niger

## Abstract

This study reports the draft genome of an *Acinetobacter baumannii* strain niger01 co-producing *bla*_NDM-1_, *bla*_OXA-23_, and *bla*_OXA-69_ genes. The genome size is 4,084,803 bp, 39.0% GC content, and harbors several insertion sequences. The emergence of new high-risk clones presents an escalating problem that significantly challenges the public health system.

## ANNOUNCEMENT

Carbapenem-resistant *Acinetobacter baumannii* is classified as an urgent threat by the United States Centers for Disease Control and Prevention and a priority-1 critical pathogen by the World Health Organization (1). It is also frequently classified based on its resistance profile as extensively drug-resistant (2, 3). In this study, we sequenced and analyzed the genome of *A. baumannii* strain niger01, isolated from a human urine sample in the National Reference Laboratory for Antimicrobial Resistance, Niamey, Niger.

In 2024, the *A. baumannii* strain niger01 was recovered from the urine of a male adult patient in Niger. The urine specimen was initially inoculated onto cystine–lactose–electrolyte-deficient agar and incubated aerobically at 37°C for 18–24 h. Distinct colonies were subsequently purified through repeated sub-culturing and their susceptibility determined through the Kirby-Bauer disc diffusion method as described by CLSI 2024. Due to its resistance to all antibiotics tested, including carbapenems, it was sent to the National Reference Laboratory for Antimicrobial Resistance for further characterization. Species identification was conducted using the Vitek 2 Compact system (bioMérieux SA, Marcy-l’Étoile, France). The isolate was thereafter sequenced at the Noguchi Memorial Institute for Medical Research, Accra, Ghana. Genomic analysis was performed as previously described (4). Genomic DNA was extracted from an overnight culture of the isolate grown statically in nutrient broth at 37°C using the QIAamp DNA Mini Kit (Qiagen, Hilden, Germany) according to the manufacturer’s instructions. Sequencing libraries were prepared with the DNA Prep (M) Tagmentation Kit (Cat. No. 20060059; Illumina Inc., San Diego, CA, USA), which includes DNA fragmentation, tagmentation, index addition, and amplification of indexed fragments. The libraries were diluted to a final concentration of 2 nM, pooled, and sequenced on the NextSeq 1000 platform (Illumina Inc.) using paired-end 2 × 150 bp chemistry. DNA concentration and fragment size distribution were assessed using the Qubit 1X dsDNA HS Assay on a Qubit 4 Fluorometer (ThermoFisher Scientific, USA) and the 2100 Bioanalyzer (Agilent Technologies, USA), respectively. Raw sequencing reads and index sequences with quality scores below Q20 were trimmed using Trimmomatic v0.39 (5), and quality control was performed with FastQC v1.0 (6). High-quality reads were assembled *de novo* using SPAdes v.3.15.3 (7), and assembly metrics were evaluated with QUAST v5.2.0 (8). Criteria for assembly quality included *Q*-scores >30, a minimum coverage of 20×, and minimum contig lengths of 200 bp. The contamination and completeness of the assembled genome were determined using CheckM2 v1.1.0 (9). Species identification was further validated by integrating results from FastANI v1.33 for pairwise average nucleotide identity analysis, and EzAAI v1.2.3 for average amino acid identity estimation, using the *A. baumannii* ATCC 19606^T^ (ASM1933165v1) reference genome as reference (10, 11). The genome was annotated using the NCBI Prokaryotic Genome Annotation Pipeline v.6.10 (12). Multi-locus sequence typing (MLST) was performed using MLST toolkit (v.2.23.0) by Seemann T, available online at https://github.com/tseemann/mlst, with default parameters. Subsequently, antibiotic resistance genes were identified using abricate v1.0.1 against the ResFinder database version 4.0 at https://github.com/tseemann/abricate (13). The summary result of the analysis is shown in Table 1. Ethical approval to conduct this study was granted by the National Ethics Committee for Health Research (CNERS), under the Ministry of Public Health of Niger, number 104/2024/CNERS.

**TABLE 1:** Assembly statistics of *A. baumannii* strain niger01 and some genomic characteristics, including resistance genes and plasmid replicons

Features	Value
Genome accession no.	JBRTYG000000000
BioSample accession no.	SAMN52836291
BioProject accession no.	PRJNA1346467
Genome completeness (%)	100
Genome contamination (%)	0.91
Read counts	7,841,518
Average nucleotide identity (%)	97.6
Average amino acid identity (%)	100
Genome coverage (×)	288×
Multilocus sequence typing	ST231
Genome size (bp)	4,084,803
*N*_50_ (bp)	134,026
GC content (%)	39.0%
Number of contigs	80
CDS	3,902
rRNA	3
repeat_region	1
tRNA	63
tmRNA	1
No. of genes	3,969
Predicted insertion sequences	IS481, IS5, ISL3, IS21, IS91, IS4, IS91, IS30, IS30, and IS6
Predicted antimicrobial resistance genes	*aac(3)-Ia*, *aadA1*, *aph(3″)-Ib*, *aph(3′)-Ia*, *aph(6)-Id*, *bla*_ADC-25_, *bla*_NDM-1_, *bla*_OXA-23_, *bla*_OXA-69_, *dfrA1*, *sul1*, *sul2*, and *tet(B*)
Disinfectant resistance genes	*qacE*

## Data Availability

Genome sequence reads have been deposited in the Sequence Read Archive under BioProject accession PRJNA1346467. The Whole Genome Shotgun project has been deposited at DDBJ/ENA/GenBank under accession number JBRTYG000000000. The version described in this paper is version JBRTYG000000000. Raw sequencing data are accessible in the SRA under accession SRR35942793.
